# Efficacy of Serotonin Type 3 Receptor Antagonist Ramosetron on Diarrhea-Predominant Irritable Bowel Syndrome (IBS-D)-Like Symptoms in Patients with Quiescent Inflammatory Bowel Disease: A Randomized, Double-Blind, Placebo-Controlled Trial

**DOI:** 10.3390/jcm11236882

**Published:** 2022-11-22

**Authors:** Toshihiko Tomita, Hirokazu Fukui, Daisuke Morishita, Sumire Mori, Tadayuki Oshima, Shinichiro Shinzaki, Hiroto Miwa

**Affiliations:** Division of Gastroenterology and Hepatology, Department of Internal Medicine, Hyogo Medical University, Nishinomiya 663-8501, Japan

**Keywords:** ramosetron, serotonin, inflammatory bowel disease, irritable bowel syndrome, double-blind method

## Abstract

Patients with quiescent inflammatory bowel disease (IBD) frequently suffer diarrhea-predominant irritable bowel syndrome (IBS-D)-like symptoms, such as abdominal pain or stool irregularities. Here, we assessed the effect of ramosetron, a serotonin type 3 (5-HT_3_) receptor antagonist, on IBS-D-like symptoms in patients with quiescent IBD. Seventy patients with quiescent IBD, who met the Rome III diagnostic criteria for IBS-D, were randomly assigned to receive either ramosetron (5 μg; n = 35) or a placebo (n = 35) orally once daily for 4 weeks. The primary endpoint was the responder rate for global assessment of relief from overall IBS-D-like symptoms. The responder rates for relief of abdominal pain/discomfort and improvement of bowel habits were also evaluated. The responder rate for relief from overall IBS-D-like symptoms at the final evaluation point was significantly higher in the ramosetron group (35.5%) than in the placebo group (11.4%) (*p* = 0.037). The responder rate for improvement of bowel habits was significantly higher in the ramosetron group (38.7%) than in the placebo group (14.3%) (*p* = 0.028). The reduction of stool frequency was significantly greater in the ramosetron group than in the placebo group (*p* = 0.044). Ramosetron is effective for relief of overall IBS-D-like symptoms in patients with quiescent IBD.

## 1. Introduction

Recent advances in the development of biologic agents have greatly improved the outlook for patients with inflammatory bowel disease (IBD), resulting in a high rate of quiescent remission [1]; thus, the number of patients with quiescent IBD worldwide has undoubtedly increased. The next important issue for these patients is improvement of their quality of life (QOL), as they do not have a QOL equivalent to that of healthy individuals [2]. In particular, patients with quiescent IBD frequently suffer symptoms such as abdominal pain or stool irregularities, which are characteristic of irritable bowel syndrome (IBS) [2,3]. If such symptoms meet the diagnostic criteria for IBS in patients with quiescent IBD, they are referred to as “IBS-like symptoms” [2,3,4], and they are present in 30–50% of such patients [2,3,5,6]. As these IBS-like symptoms in quiescent IBD significantly disturb health-related QOL [5], their treatment is a serious issue.

Ramosetron, which is a potent and selective serotonin type 3 (5-HT_3_) receptor antagonist, has been evaluated for its efficacy and safety in the treatment of diarrhea-predominant IBS (IBS-D) [7,8,9,10,11]. Although ramosetron is not yet available for the treatment of IBS-D in Western countries, several randomized controlled trials (RCT) from Japan have demonstrated consistently that ramosetron is significantly more effective than placebo for treating IBS-D, and that it has no significant safety concerns [12,13]; accordingly, ramosetron is used for treatment of IBS-D in Japan and some Asian countries, and has been shown to relieve abdominal pain or stool irregularity [12,13]. In the present randomized, double-blind, placebo-controlled study, we investigated the effectiveness of ramosetron for treatment of IBS-D-like symptoms in patients with quiescent IBD.

## 2. Materials and Methods

### 2.1. Patients

Patients who had quiescent IBD with IBS-D-like symptoms were enrolled into this randomized, double-blind, placebo-controlled trial, at our university hospital, from January 2016 to June 2019. The study protocol was designed in accordance with the Declaration of Helsinki, approved by the Ethics Committee of Hyogo Medical University (Approval No. 2075) and registered in the University Hospital Medical Information Network (registration number UMIN 000023399).

Quiescent Crohn’s disease (CD) was defined as a CD activity index (CDAI) of ≤150 with a C-reactive protein (CRP) level of ≤0.3 mg/dL [4,14]. Quiescent ulcerative colitis (UC) was defined as a clinical activity index (CAI) of ≤4 with a CRP level of ≤0.3 mg/dL [3,15]. As we did not have a validated Japanese version of the Rome IV diagnostic questionnaire, we used a Japanese version of the Rome III diagnostic questionnaire for functional gastrointestinal disorders, to evaluate IBS-D-like symptoms [16,17]. As all the patients had an organic disease such as IBD, they were not real IBS patients; however, when their symptoms met the criteria for the diagnosis of IBS-D, the patients were defined as having IBS-D-like symptoms. Patients satisfying the inclusion and exclusion criteria (Supplementary Table S1) were monitored for a week, to ensure that their abdominal symptoms and stool frequency met the criteria. All the patients provided their written informed consent, before participating in this study.

### 2.2. Study Design

**Figure 1:**
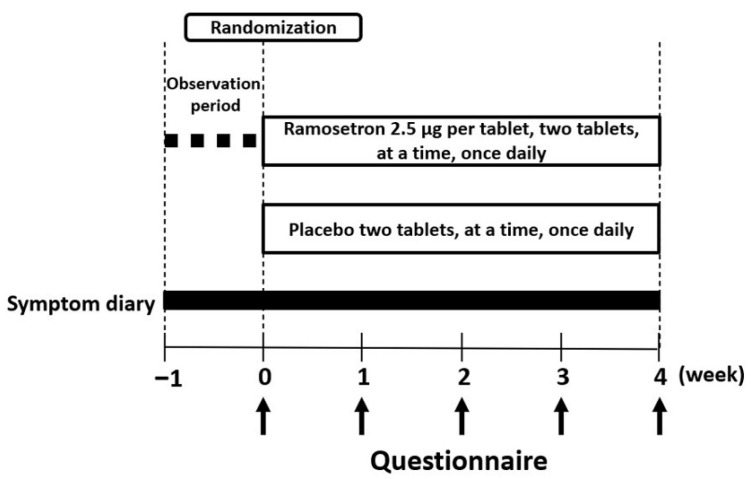
Study design.

**Figure 2:**
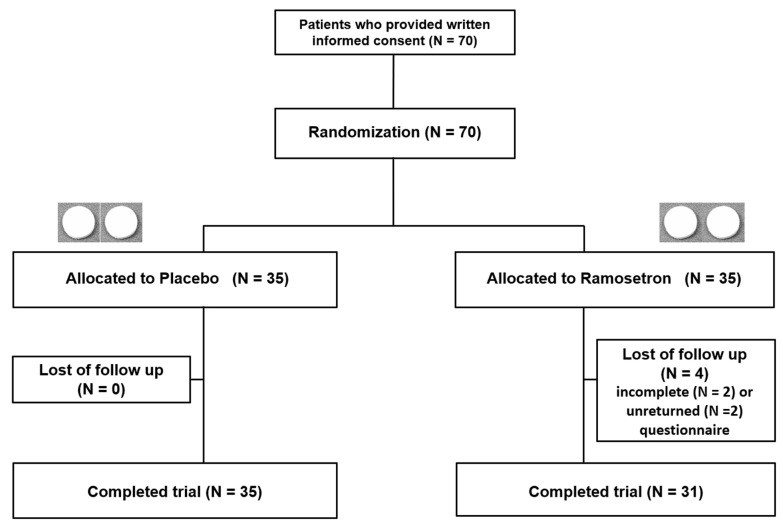
Flowchart of patients throughout the study period. Details of patients who dropped out are shown.

This randomized, double-blind, placebo-controlled clinical study comprised a 1-week provisional registration period and a 4-week treatment period (Figure 1). After a 1-week baseline period, 70 eligible patients, who met the definitions of quiescent CD/UC with IBS-D-like symptoms, were finally enrolled. The enrolled patients were assigned randomly to 4 weeks of oral treatment with placebo (n = 35) or ramosetron (n = 35, 5.0 μg) once daily before breakfast (Figure 2). In the present study, a third party, with no affiliation to this research, assigned the study drugs, using a pre-generated assignment list with consecutive numbers from 1 to 70. Computer-generated randomization sequences were prepared using a permuted block method (block size, 10) without stratification. All the drugs were prepared as the tablet form of a set size that could not be identified from exterior appearance. A master list linking subject ID numbers to subjects was securely managed and stored by the investigator in charge of the study, and unmasking was not allowed unless specific procedures were followed.

### 2.3. Data Collection, Efficacy and End Points

Demographic data including age, gender, BMI, type of IBD, duration of IBD, smoking and medication were collected. During the baseline and treatment periods, the patients recorded their IBS symptoms, including abdominal pain, bowel habits or stool frequency, on a diary paper file every day.

Every 7 days during the treatment, the patients self-assessed their relief of overall IBS symptoms, abdominal pain/discomfort and improvement in bowel habits compared to the baseline period, and graded their responses using a 5-point ordinate scale (0, completely relieved; 1, considerably relieved; 2, somewhat relieved; 3, unchanged; and 4, worsened). Patients with scores of 0 or 1 at each weekly evaluation point were defined as weekly responders. Patients who were weekly responders for at least 2 of the 4 weeks in a month were defined as responders at the final evaluation point.

The primary end point was the responder rate for global assessment of relief from overall IBS-D like symptoms at the final evaluation point.

The secondary end points were the responder rates for relief of abdominal pain/discomfort and improvement of bowel habits at the final evaluation point. Other secondary end points were the weekly responder rates for global assessment of relief from overall IBS symptoms, abdominal pain/discomfort and improvement of bowel habits, and changes in the weekly stool frequency average.

### 2.4. Statistical Analysis

We had preliminarily investigated the effect of ramosetron (5.0 μg once daily) in 20 patients with quiescent CD who had IBS-D-like symptoms, and we found that 14 (70%) of them had been responders to global assessment of relief from overall IBS symptoms at the final evaluation point, following 4 weeks of treatment [18]. Furthermore, in a phase III trial of ramosetron for patients with IBS-D, the responder rates of relief from overall IBS symptoms were 32.0% and 50.7% in the placebo and ramosetron groups, respectively, [8]. Overall, we set the responder rates of relief from overall IBS symptoms in the placebo and ramosetron groups at 30% and 65%, respectively. When the add-on effect was set at 35%, with a 1-sided level of significance of 5% and a power of 80%, the calculation revealed that the required sample size was 35 subjects (31 subjects plus 4 dropouts) in each group.

All results were expressed as mean ± standard deviation (SD). The paired t-test, the Mann–Whitney *U* test, Fisher’s exact test and analysis of covariance (ANCOVA) were used for comparison of the two groups. Statistical significance was defined as a value of *p* < 0.05. Statistical analysis was performed using JMP for Windows (Version 14; JMP Pro, SAS Institute Inc., Cary, NC, USA).

## 3. Results

### 3.1. Enrolment and Baseline Characteristics of the Patients

Figure 2 shows a flowchart of the patients’ conditions. The 70 patients with quiescent IBD showing IBS-D-like symptoms were enrolled and randomly assigned to either the placebo (n = 35) or the ramosetron (n = 35) groups. In the ramosetron group, 4 patients dropped out: 2 of those patients did not complete the questionnaire, and the other 2 patients did not return it; thus, the analyses were performed using data from 35 placebo-treated and 31 ramosetron-treated patients.

The demographic and baseline characteristics of all the randomized patients are shown in Table 1: there were no significant differences between the two groups in regard to those characteristics, including age, gender, BMI, disease duration, smoking, medication, CRP and CDAI/CAI. No significant adverse events were encountered.

### 3.2. Effect of Ramosetron on Overall IBS-D-Like Symptoms in Patients with Quiescent IBD

**Figure 3:**
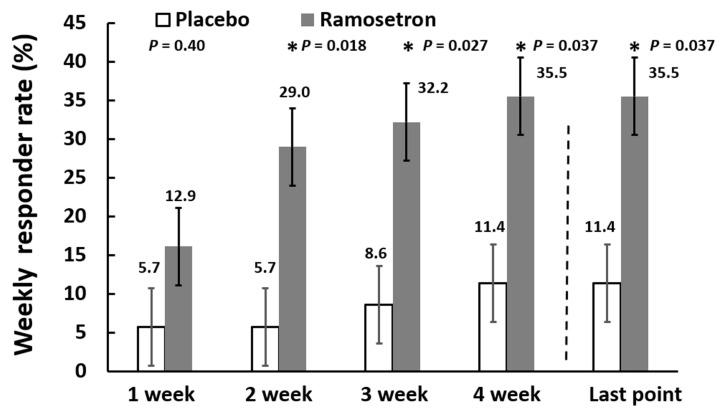
Weekly responder rates for relief of overall IBS-D-like symptoms. Results are expressed as mean ± SD. * *p* < 0.05 vs. placebo.

Weekly responder rates for relief of overall IBS-D-like symptoms gradually increased up to 35.5% in the ramosetron group, whereas they remained below 11.4% in the placebo group during the experimental period (Figure 3). The responder rates were significantly higher in the ramosetron group than in the placebo group at 2, 3 and 4 weeks from the start of treatment. As for the primary end point, the responder rate for relief of overall IBS-D-like symptoms at the final evaluation point was significantly higher in the ramosetron group (35.5%) than in the placebo group (11.4%).

### 3.3. Effect of Ramosetron on Abdominal Discomfort/Pain and Abnormal Bowel Habits in Patients with Quiescent IBD

**Figure 4:**
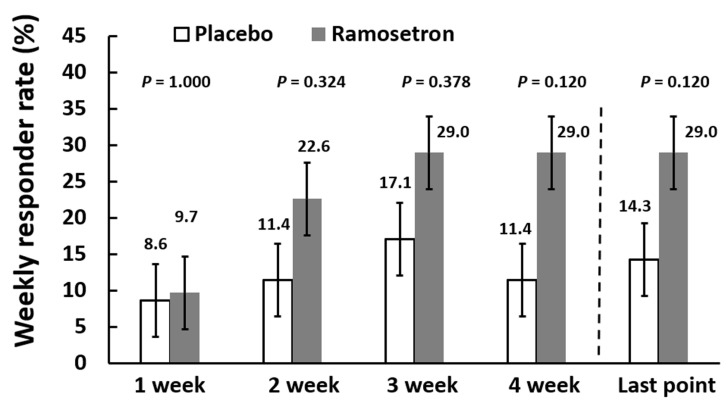
Weekly responder rates for relief of abdominal discomfort/pain. Results are expressed as mean ± SD.

For the secondary end points, we investigated the responder rates for relief of abdominal pain/discomfort and improvement in bowel habits at the final evaluation point. Weekly responder rates for relief of abdominal discomfort/pain gradually increased up to 29.0% in the ramosetron group, but no significant differences were evident between the ramosetron and placebo groups (Figure 4); thus, the responder rate at the final evaluation point did not differ significantly between the two groups (Figure 4).

**Figure 5:**
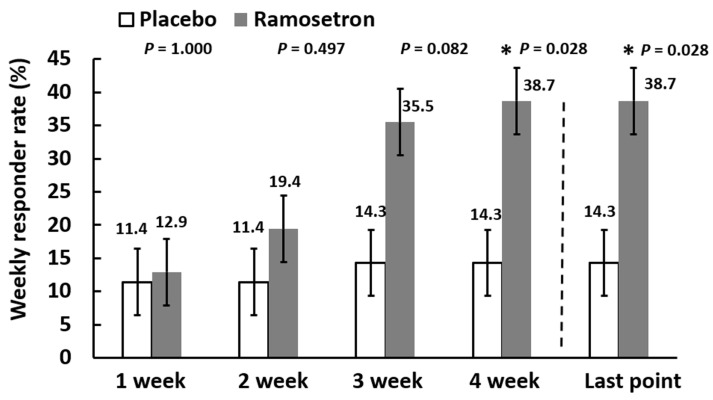
Weekly responder rates for improvement of bowel habits. Results are expressed as mean ± SD. * *p* < 0.05 vs. placebo.

Weekly responder rates for improvement of bowel habits gradually increased up to 38.7% in the ramosetron group, whereas they remained below 14.3% in the placebo group during the experimental period (Figure 5). The responder rate at the final evaluation point was significantly higher in the ramosetron group (38.7%) than in the placebo group (14.3%).

### 3.4. Effect of Ramosetron on Weekly Changes in Stool Frequency

**Figure 6:**
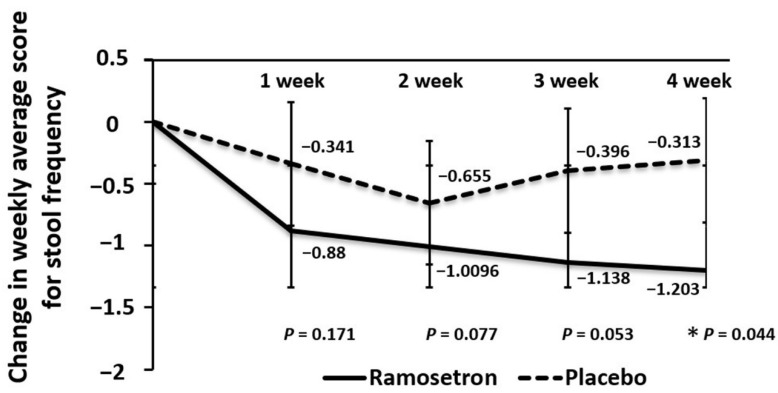
Weekly changes in stool frequency. Results are expressed as mean ± SD. * *p* < 0.05 vs. placebo.

The reduction of stool frequency remained greater in the ramosetron group than in the placebo group during the experimental period (Figure 6). At the 4-week point from the start of treatment, the reduction was significantly greater in the ramosetron group than in the placebo group.

## 4. Discussion

It has been reported that patients with quiescent IBD frequently suffer from IBS-D-like symptoms [2,3,5,6], and that such symptoms significantly worsen QOL [5]. Therapeutic strategies for these IBS-D-like symptoms have yet to be established. Ramosetron, a 5-HT_3_ receptor antagonist, was shown to be effective for the treatment of IBS-D in several clinical trials [7,8,9,10,11], although it had previously only been used as a postoperative or post-chemotherapy antiemetic [19,20,21]. The present study is the first trial to have investigated the effectiveness of ramosetron for treatment of IBS-D-like symptoms in patients with quiescent IBD, and we were able to show clearly that ramosetron was indeed significantly effective for overall relief of IBS-D-like symptoms.

IBS-D-like symptoms include abnormal bowel habits and frequent defecation: in this context, we investigated the effect of ramosetron on each symptom by sub-analyses. The results indicated that ramosetron was effective for improving bowel habits and reducing stool frequency in patients with quiescent IBD. Although the mechanism by which ramosetron ameliorates diarrhea remains unclear, its inhibitory effect on 5-HT_3_ receptors in the myenteric plexus and vagal afferent neurons is thought to play a pivotal role in the normalization of intestinal motility [22]. It has been reported that activated 5-HT signaling via 5-HT_3_ receptors exaggerates colonic motility in IBS-D patients [23]. Furthermore, ramosetron has been shown to reduce stress-induced diarrhea and defecation in a rat model [24]. The mechanism by which ramosetron helps to relieve overall IBS-D-like symptoms in quiescent IBD is still unclear; however, it is tempting to speculate that inhibition of 5-HT signaling in the intestinal tract contributes to improvement of intestinal motility.

IBS-D-like symptoms also include abdominal pain/discomfort. This study found a strong tendency for ramosetron to protect against abdominal pain/discomfort in patients with quiescent IBD, although the effect was not statistically significant: one possible reason may have been the relatively short (4 weeks) duration of treatment with ramosetron; longer treatment would perhaps have yielded a significant effect. It is widely accepted that psychological stress plays a pivotal role in the pathophysiology of IBS-D in humans, as well as experimental animals [25,26]. Accordingly, subjective symptoms such as abdominal pain/discomfort are likely affected by psychological conditions. Interestingly, patients with not only IBS-D but also IBD with IBS-D-like symptoms show a significantly worse anxiety score [3,5]: in this context, it is noteworthy that 5-HT_3_ receptor antagonists may be useful for treatment of anxiety disorders, although conflicting data have also been reported [27]. In a future study, we would like to evaluate changes of mental status after ramosetron treatment in patients with quiescent IBD who have IBS-D-like symptoms.

In summary, this is the first reported study to have investigated the effectiveness of ramosetron for relief of overall IBS-D-like symptoms in patients with quiescent IBD. We found that ramosetron was effective for improving bowel habits and reducing stool frequency; however, a major study limitation was that the data were obtained after only a short treatment period at a single institution. In the pathophysiology of IBS-D, interaction of various factors (visceral sensitivity, bowel motility, gut microbiome, mucosal immunity, psychological stress, etc.) may be involved [26]. As for IBD patients, the decreased abundances of *Faecalibacterium* and *Fusicatenibacter*, which respectively promote the production of butyrate and IL-10, have been repeatedly demonstrated as significant alterations of gut microbiome [28,29,30]; however, little is known about the underlying pathophysiology in patients with quiescent IBD suffering IBS-D-like symptoms, and it may be quite different from that in IBS patients without IBD. Interestingly, IBS frequently develops during the healing stage of infectious colitis [31], and minimal inflammation of intestinal tissues has recently been highlighted as a key player in the pathophysiology of IBS-D [26,32]. On the other hand, minimal inflammation certainly persists in the intestinal tissues of patients with quiescent IBD [4,6]. These findings suggest that intestinal minimal inflammation is a common factor in the pathophysiology of not only IBS-D but also quiescent IBD associated with IBD-like symptoms: therefore, it would be interesting to investigate inflammation-related aspects in such patients during treatment with 5-HT_3_ receptor antagonists.

## Figures and Tables

**Table 1:** Demographics and baseline characteristics.

	Placebo	Ramosetron	*p* Value
Number	35 (CD: 31, UC: 4)	31 (CD: 25, UC: 6)	
Age (year)	44.5 ± 9.9	46.6 ± 12.4	0.589
Gender, male (n)	29 (82.8%)	26 (83.9%)	1.00
BMI (kg/m^2^)	20.9 ± 3.0	21.6 ± 3.5	0.550
Type of CD			
L1 (ileal)	9	6	
L2 (colonic)	5	5	
L3 (ileocolonic)	17	14	
Type of UC			
Proctitis	0	4	
Left-sided	2	1	
Total colon	2	1	
Duration of disease (year)	17.2 ± 7.6	19.9 ± 9.9	0.291
Smoking (n)	13 (37.1%)	14 (45.2%)	0.618
Medication			
5-ASA/sulfasalazine	20	21	
Prednisolone	0	0	
Azathioprine/6-MP	1	2	
Anti-TNF-α antibody	18	14	
CRP	0.2 ± 0. 3	0.2 ± 0. 3	0.810
CDAI	68.2 ± 40.9	73.4 ± 3.3	0.639
CAI	0.25 ± 0.5	0.83 ± 0.7	0.239

Data are expressed as mean ± SD. CD, Crohn’s disease; UC, ulcerative colitis; CDAI, CD activity index; CAI, UC clinical activity index; 5-ASA, 5-aminosalicylate; 6-MP, 6-mercaptopurine; TNF, tumor necrosis factor; CRP, C-reactive protein; CDAI, CD activity index; CAI, clinical activity index.

## Data Availability

Any data referred to in this work will be available on request.

## Supplementary Materials

The following supporting information can be downloaded at https://www.mdpi.com/article/10.3390/jcm11236882/s1, Supplementary Table S1: Inclusion and exclusion criteria.
