# Supplementary material for: Efficacy of Serotonin Type 3 Receptor Antagonist Ramosetron on Diarrhea-Predominant Irritable Bowel Syndrome (IBS-D)-Like Symptoms in Patients with Quiescent Inflammatory Bowel Disease: A Randomized, Double-Blind, Placebo-Controlled Trial
Source: J Clin Med. 2022 Nov 22;11(23):6882. doi: 10.3390/jcm11236882 (PMC9736938; doi:10.3390/jcm11236882)
Supplement: Supplementary file 1 [file jcm-11-06882-s001.zip › jcm-2030693-supplementary.pdf]

Supplementary Table S1. Inclusion and exclusion criteria

Diarrhea predominant irritable bowel syndrome (IBS-D)

- IBS-D is defined by recurrent abdominal pain/discomfort for at least 3 days per month in the preceding 3 months, in association with two or more of the following: improved defecation, onset associated with a change in the frequency of stools, and/or onset associated with a change in the form (appearance) of stools. Furthermore, patients had loose (mushy) or watery stools at least 25% of the time and hard or lumpy stools for less than 25% of bowel movements.

- Patients were eligible if they fulfilled the criteria for the last 3 months, with symptom onset at least 6 months prior to diagnosis.

- Organic diseases were excluded by colonoscopy or double-contrast barium enema if these examinations had not been performed within 5 years.

- Patients satisfying the inclusion and exclusion criteria for typical IBS-D symptoms during a 1-week baseline period were enrolled.

Quiescent inflammatory bowel disease

- Quiescent Crohn's disease (CD) and ulcerative colitis (UC) were defined as follows: quiescent CD: Crohn's disease activity index $\leq$ 150 and CRP $\leq$ 0.3; quiescent UC: clinical activity index $\leq$ 4 and CRP $\leq$ 0.3.
- The following patients were excluded:

1. Patients <20 years old or ≥70 years old
2. Patients with a past history or current evidence of ischemic colitis
3. Patients with concurrent infectious colitis
4. Patients with hyperthyroidism, hypothyroidism
5. Patients with malignant tumors
6. Patients with current evidence of severe depression or a severe anxiety disorder
7. Patients with concurrent serious cardiovascular, respiratory, renal, hepatic, gastrointestinal (excluding IBS), hematological, or neurological/psychiatric diseases
8. Patients with a history of drug allergies
